# Next-generation vaccines are showing promise against glioblastoma

**DOI:** 10.18632/oncotarget.28636

**Published:** 2024-08-05

**Authors:** Robert O. Dillman, Daniela A. Bota

**Keywords:** glioblastoma, vaccines

Glioblastoma (GBM) is a rare but highly lethal malignancy that occurs almost entirely in adults [1]. Annually, there are about 13,000 new GBM diagnoses in the United States and about 12,000 deaths. For many years, GBM has been considered synonymous with Grade IV astrocytoma, though, recently, the IDH mutant Grade IV astrocytoma has been considered a separate and distinctive pathology [2]. By this definition, GBM accounts for about 15% of primary brain tumors and 50% of adult gliomas and is by far the most lethal [3]. Median age at diagnosis is 64 years; most patients are diagnosed in the 6th to 8th decades of life. Standard combination therapy (maximum extent of surgical resection followed by combined radiation and temozolomide chemotherapy) is not attempted in many patients because of age-associated co-morbidities [4]. In fact, historically, the median age in GBM clinical trials with a control arm of standard aggressive therapy was only 54 to 59 years [5–15]; it is only recently that some randomized trials have had a median age as high as 60 years [16–18].

Unfortunately, even in the more favorable populations enrolled in clinical trials, standard therapy is associated with poor outcomes: median progression-free survival (PFS) of about 7 months, median overall survival (OS) of 16 months, 1-year OS of 70%, and 2-year survival of 25% when calculated from enrollment just prior to concurrent involved-field radiation therapy and temozolomide chemotherapy (RT/TMZ) [5–9, 12]. When attempting to compare survival outcomes between GBM clinical trials, it is important to recognize the date of enrollment from which PFS and OS are calculated and to appreciate differences in eligibility criteria that may limit data to specific subsets of patients. Trials with an enrollment date after completion of RT/TMZ have inclusion/exclusion criteria that eliminate many higher-risk patients [7, 10, 11, 14]. In addition, for various reasons, some trials have enrolled only a subset of GBM patients based on specific tumor cell phenotypes and genotypes [7, 11, 14, 17, 18].

There has been little therapeutic progress against GBM during the past 20 years since the landmark 2005 publication that established concurrent RT/TMZ as standard treatment following surgical resection in patients with newly diagnosed primary glioblastoma with good performance status [5]. Numerous randomized trials have failed to demonstrate an improvement in survival from enrollment prior to starting concurrent RT/TMZ (Table 1) [5–9, 12, 17, 18]. The original Stupp study also established 6 cycles of TMZ post-RT/TMZ as standard maintenance or consolidation therapy for patients who had recovered following concurrent RT/TMZ [5]. Subsequent randomized trials attempting to build on this RT/TMZ—TMZ platform failed to show an increase in PFS or OS for dose-dense TMZ [6],or extending TMZ beyond six 28-days cycles [16], but were associated with greater toxicity. Only one clinical trial, which added alternating tumor-treating fields (TTF) to TMZ, has demonstrated a survival benefit compared to TMZ alone in patients who have recovered from RT/TMZ [10], but that trial limited enrollment to patients without evidence of progressive disease (PD) or pseudoprogressive disease (PsPD) following RT/TMZ, which typically excludes about 20% of patients who started RT/TMZ [19]. Other randomized trials enrolling patients after RT/TMZ have failed to demonstrate a survival benefit compared to TMZ alone (Table 2) [6, 10, 11, 14, 16, 20, 21], although one randomized trial, which was negative for its original PFS primary objective was positive for OS compared to pooled data derived from standard treatment arms of five randomized trials [21].

**Table 1:** Randomized trials of therapies in patients with GBM enrolled prior to concurrent radiation therapy and temozolomide

Product	Class	Key eligibility and exclusions	Report	Patients numbers	Results
Temozolomide TEMODAR	Chemotherapy	Age 18–70 yrs	Stupp 2005	573 (1:1) Primary	21% increase OS for RT plus TMZ
Temozolomide TEMODAR	Dose-dense chemo		Gilbert 2013	833 (1:1)	No increase PFS or OS, more toxicity
Bevacizumab AVASTIN	Anti-VEGF Moab		Gilbert 2014	637 (1:1) Primary	Better PFS, same OS
Bevacizumab AVASTIN	Anti-VEGF Moab		Chinot 2014	921 (1:1) Primary	Better PFS, same OS
Cilengitide	Small molecule, Targets alpha-integrins, anti-angiogenesis	Methylated *MGMT* promoter	Stupp 2014	545 (1:1)	No increase PFS or OS
Audencel	DC + autologous whole tumor lysate	Age 18–70 yrs >70% resection	Buchroithner 2018	76 (1:1)	No difference PFS or OS
Depatuxizumab Mafodotin	Anti-EGFR antibody conjugate	EGFR-amplified	Lassman 2019	639 (1:1) Primary	No increase PFS or OS
Nivolumab	Anti-PD-1	Methylated *MGMT* promoter	Lim 2022	485 1:1	No increase PFS or OS
Marizomib	Proteosome inhibitor		Roth 2024	749 (1:1)	No increase OS or PFS

Abbreviations: DC: dendritic cell; EGFR: epidermal growth factor receptor; OS: overall survival; PD: progressive disease; PD-1: programmed death molecule-1; PFS: progression-free survival; RT: radiation therapy; TMZ: temozolomide; VEGF: vascular endothelial growth factor.

**Table 2:** Trials of therapies in patients with GBM who were randomized after recovery following concurrent RT/TMZ

Product	Class	Key eligibility and exclusions	Report	Number patients	Results
Temozolomide TEMODAR	Dose-dense chemo	Enrolled prior to RT/TMZ, randomized after	Gilbert 2013	833 (1:1)	No increase PFS or OS but increased toxicity
Tumor Treating Fields (OPTUNE)	Electrical field device	PD/PsPD IE	Stupp 2017	695 (2:1) Primary	31% increase OS post RT/TMZ
Rindopepmut With KLH plus GM-CSF	Peptide vaccine to EGFRvIII-mutated peptide, conjugated to KLH + GM-CSF	EGFRvIII expressed PD/PsPD IE	Weller 2017	745 (1:1)	No increase PFS or OS
ICT-107	DC vaccine, 6 TAA peptides	HLA-A2+ PD/PsPD IE	ICT PR 2017	414 (1:1)	Suspended
ICT-107	DC vaccine, 6 TAA peptides	HLA-A1 or HLA-A2+ PD/PsPD IE	Wen 2019	124 (2:1)	Increased PFS; no increase OS
Temozolomide TEMODAR	12 vs. 6 TMZ cycles	PD/PsPD IE	Balana 2020	159 (1:1)	No increase PFS or OS but increased toxicity
DCVax-L	DC vaccine, whole tumor lysate	Age 18–70 years PD/PsPD IE	Liau 2018, 2022	331 (2:1)	No increase PFS; increase OS vs. historical control

Abbreviations: DC: dendritic cell; EGFR: epidermal growth factor receptor; GM-CSF: granulocyte-macrophage colony stimulating factor; IE: ineligible; KLH: keyhole limpet hemocyanin; OS: overall survival; PD: progressive disease; PFS: progression-free survival; PR: Press Release; PsPD: pseudoprogressive disease; RT: radiation therapy; TAA: tumor-associated antigens; TMZ: temozolomide.

Two large, randomized trials tested the addition of treatment with the monoclonal antibody bevacizumab, which blocks vascular endothelial growth factor (VEGF) [8, 9]. In both trials, bevacizumab was associated with increased PFS but not OS and was associated with increased toxicity. The apparent improvement in PFS may be an artifact of decreased blood flow and decreased peritumoral edema, which results in delays in MRI-based diagnosis of disease progression [19]. However, many neuro-oncologists continue to use this regimen because of the prolonged PFS and the beneficial effects on cerebral edema, which may permit avoiding having to use high-dose corticosteroids. Based on randomized clinical trial results, standard maintenance regimens post RT/TMZ include TMZ alone [5–11, 15–18], TMZ with bevacizumab (in selected cases where the patients require high amounts of steroids) [8, 9], and TMZ with alternating electronic tumor-treating fields (TTF, Optune^®^) [10].

Vaccines have been considered a promising approach for GBM for many years [22]. All have been well-tolerated, with the most common adverse events being local injection reactions and various “flu-like” symptoms that are mild to moderate in severity, brief in duration, and generally self-limited in that medical treatment generally is not required. However, efficacy has not been demonstrated in randomized trials (Tables 1 and 2) [11, 12, 14, 20, 21]. Two basic strategies have been used. The first approach is vaccination against antigenic targets shared by a subset of GBM patients (rindopepimut, ICT-07). Rindopepimut includes a mutated EGFR-receptor peptide, expressed in about 30% of GBM, that is conjugated to keyhole limpet hemocyanin (KLH), a very immunogenic protein from mollusks, as a foreign immune-stimulating molecule, and admixed with granulocyte-macrophage colony-stimulating factor (GM-CSF) as an adjuvant to enhance the local immune response, and injected intradermally (i.d.). ICT-107 consists of autologous DC loaded with six synthetic Human Lymphocyte Antigen-A (HLA-A) restricted peptide antigens commonly shared among GBM samples and often expressed on GBM stem cells [14]. The six peptides are melanoma associated antigen (MAGE-1), human epidermal growth factor receptor-2 (HER-2), the antigen isolate from immunoselected melanoma-2 (AIM-2), tyrosine-related protein-2 (TRP-2), glycoprotein 100 (gp100) and interleukin13 receptor alpha2 (IL13Ra2). The second approach is vaccination with autologous tumor antigens (Audencel and DC-VAX-L). DCVax-L is a personal, patient-specific vaccine that consists of autologous DC incubated with a lysate of autologous tumor resected at the time of surgery that is injected i.d [13, 21]. Audencel is also a patient-specific DC vaccine loaded with antigens from whole tumor lysate, but it was injected directly into inguinal lymph nodes [12].

DCVax-L has yielded the most promising results among these vaccine products [21] Each vaccine dose was based on incubating 2.5 million DC with a lysate of autologous tumor before cryopreservation. The DC-vaccine was injected i.d. without an adjuvant on days 0, 10, and 20, then months 2, 4, and 8, and then every 6 months starting at month 12. The DCVax-L phase 3 trial was complicated by enrollment and study design. Patients were enrolled between 2007 and 2009, then paused for economic reasons during 2009 and 2011, then resumed, and enrollment finally closed in 2015 after 331 of the planned 348 patients had been enrolled [13, 21, 23]. Obtaining a sufficient quantity of tissue at the time of surgery was a major challenge, and sufficient monocytes to differentiate into DC could not be obtained from many patients. Furthermore, patients were excluded from treatment if they had PD or PsPD on MRI scans obtained after RT/TMZ. Consequently, only 331 of 1,599 screened patients (21%) were randomized. The trial was double-blinded, and the original design had a 2:1 randomization to DCVax-L or autologous monocyte placebo control. Unfortunately, patients in the control arm were allowed cross-over to receive DCVax-L at the time of progressive disease, even though patients and physicians remained blinded to the treatment being administered. Therefore, the primary objective was PFS, which relied heavily on the interpretation of magnetic resonance imaging (MRI) scans using RANO criteria (response assessment in neuro-oncology) [24–26], rather than OS since DCVax-L was administered to 64 of the 99 patients in the control arm. Based on the original design of intent-to-treat analysis per randomization after RT/TMZ, the trial was negative with a median PFS of 6.2 months for DCVax-L vs. 7.6 months for placebo (*p* = 0.47) [23]. However, as the trial progressed, the limitations of relying on RANO for interpretation of disease control became apparent as some patients appeared to develop PsPD during treatment, as might be expected if a vaccine was causing inflammation in the GBM tumor bed. Therefore, the statistical analysis plan was changed to compare OS in the DCVax-L arm to a historical control cohort consisting of patients treated with standard maintenance TMZ in control arms of five published randomized trials [6, 8, 10, 11, 14]. In this analysis, from the date of randomization, the median OS for the 232 patients randomized to DCVax-L was 19.3 months versus 16.5 months for the historical controls (*p* = 0.002) with a 2-year OS of about 35% and 3-year OS of 20% [21]. These results are considered encouraging and support more investigation into approaches with autologous DC delivery of autologous tumor antigens.

In the past two years, two additional promising vaccine candidates have emerged, both of which were highlighted at the 5th Glioblastoma Vaccine Summit held in Boston in March 2024. One vaccine targets a shared tumor-associated antigen; the other targets autologous antigens. SurVaxM is a peptide vaccine that targets “survivin,” a protein that is expressed on 1% to 40% of GBM cells from patients [27]. The vaccine has 3 components: [1] the tumor antigen “survivin” (BIRC5) peptide, an oncofetal tumor-associated antigen that is often expressed in cancer cells but rarely expressed in normal cells, [2] KLH, and [3] Montanide ISA-51, an immunogenic oil-in-water emulsion similar to incomplete Freund’s adjuvant. SurVaxM is injected s.c., and at the same time, 100 mg GM-CSF is injected s.c. nearby as an additional adjuvant. A phase 2 trial enrolled only newly diagnosed GBM patients who had undergone gross total resection of their primary tumors and were doing well after completing RT/TMZ [27]. The trial excluded many patients with a poor prognosis, including any patient who: (1) did not have a gross total resection based on post-op MRI scan, (2) appeared to have PD following RT/TMZ, (3) was receiving doses greater than 4 mg of the corticosteroid dexamethasone, (4) were unable to receive the first 4 injections (given every 2 weeks for 8 weeks), (5) were considered too ill to proceed with maintenance TMZ, and (6) excluded patients who would have been treated with anti-vascular endothelial growth factor (VEGF) bevacizumab or alternating electronic tumor treating fields (TTF, Optune^®^) in addition to maintenance TMZ. Patients were enrolled after RT/TMZ, and typically started treatment about three months after the original diagnosis. After the initial 4 vaccinations over 8 weeks, vaccination was continued every 12 weeks until PD. Patients were not considered evaluable unless they had remained disease-free for five months following surgery, which included the two months of vaccination. This subset of 63 patients had a median PFS of 11.4 months and a median OS of 25.9 months from the date of diagnosis by surgical biopsy [27]. The generalizability of this SurVaxM data is difficult due to the patient selection criteria that almost certainly resulted in the treatment of patients with a better prognosis given that 114 of the 178 patients screened (64%) were excluded even though screening and enrollment did not take place until after RT/TMZ. The real potential of this “off-the-shelf” product will become apparent after analysis of a multicenter randomized phase 2 trial, which was due to complete accrual in the spring of 2024 (NCT04978727).

The second promising vaccine candidate that has recently emerged is AV-GBM-1, a personal, patient-specific vaccine consisting of autologous dendritic cells that have been incubated with a lysate of autologous irradiated GBM cells that are self-renewing in short-term cell culture [28]. Such cells have features of GBM stem cells and early progenitor cells [29–31]. AV-GBM-1 differs from DC-Vax-L in that the antigen source is a pure culture of self-renewing GBM cells rather than a mix of undifferentiated and terminally differentiated GBM cells, and normal hematopoietic, vascular, stromal, and immune cells; therefore, it should potentially be a better antigen source. Also, AV-GBM-1 has been given admixed in GM-CSF, which may have desirable immune adjuvant effects and support the viability and function of the antigen-loaded DC [32–34]. Both of these patient-specific autologous DC products have similar logistical challenges that are not obstacles for off-the-shelf products. These include (1) obtaining consent and acquiring fresh tumor at the time of diagnosis, (2) obtaining a sufficient quantity of tumor protein or cells in order to manufacture the vaccine, (3) collecting sufficient numbers of monocytes for differentiation into DC in cell culture, (4) successful differentiation of monocytes into DC, (5) antigen loading of DC, and (6) overcoming complex logistics that include timely communication and coordination and shipping frozen samples to various sites for thawing and injection. Consequently, a major concern is the attrition of patients from the time of initial consent at a time when it is not even certain that the diagnosis is GBM. Table 3 summarizes patient attrition for these two products subsequent to obtaining initial consent before surgery [13, 28]. The exclusion of patients for PD or PsPD was an eligibility criteria for DCVax-L but not for AV-GBM-1. As can be seen, the successful manufacturing of DC-Vax-L was lower because of problems with intermediate products, including inadequate amount of tumor lysate, unsuccessful leukapheresis procedures, and failure during manufacturing of the final product. Ultimately the proportions of patients enrolled from those initially screened were 60/106 (56.6%) for consent at the surgery to screening and enrollment pre-RT/TMZ for AV-GBM-1, and 331/1599 (20.7%) for consent at the surgery to screening and enrollment after RT/TMZ for DCVax-L [13, 28].

**Table 3:** Attrition of patients in trials of patient-specific DC and autologous tumor antigens

	DCVax-L	AV-GBM-1
Initial consent prior to surgery	1599	106
Not enrolled for treatment/randomization	1268 (79.3%)	46 (43.4%)
Enrolled to treat of all consented prior to surgery	331 (20.7%)	60 (56.6%)
Reasons patients did not enroll for vaccine treatment		
Not Glioblastoma	306 (19.2%)	15 (14.2%)
Progression or Pseudoprogression	250 (15.6%)	0 (0%)
Insufficient tumor lysate	201 (12.6%)	2 (1.9%)
Declined before leukapheresis	121 (7.6%)	3 (2.8%)
General inclusion/exclusion criteria	91 (5.7%)	5 (4.7%)
Manufacturing product/placebo	75 (4.7%)	2 (1.9%)
Unsuccessful leukapheresis	61 (3.8%)	2 (1.9%)
Clinical Deterioration	41 (2.6%)	6 (5.7%)
Declined/Withdrew Consent	40 (2.5%)	8 (7.5%)
Investigator Decision	35 (2.2%)	4 (3.8%)
Surgery not done/processed	35 (2.2%)	4 (3.8%)
Other/unknown	12 (0.8%)	0 (0%)

Patients were enrolled for intent-to-treat with AV-GBM-1 prior to initiating RT/TMZ, but vaccination did not begin until after recovery following RT/TMZ, which typically was about three months after diagnosis. Each AV-GBM-1 dose was suspended in 500 mg GM-CSF prior to injections at weeks 1, 2, 3, 8, 12, 16, 20 and 24. After the first three weekly injections, neuro-oncologists had the option of adding concurrent treatment with any standard TMZ regimen, including TMZ alone, TMZ plus bevacizumab, or TMZ plus TTF. Eight patients were considered too ill to receive TMZ concurrently with the vaccine. All patients enrolled before concurrent RT/TMZ were considered evaluable. The 60 patients had a median PFS of 10.4 months and OS of 16.0 months from enrollment, with a 2-year OS of 33% and a 3-year OS of 23% [35]. A possible explanation for the poor correlation between PFS and OS in this trial is that AV-GBM-1 was discontinued after eight injections over six months at a time point about eight months from enrollment [36]. In subset analyses for various prognostic markers, such as O6-methylguanine-methyltransferase (*MGMT*) methylation, isocitrate dehydrogenase (*IDH*) mutation, age, concurrent dexamethasone dose, and significant central nervous system adverse events, OS curves did not separate until 2 to 3 months after vaccination was discontinued. For patients who received at least one dose of TMZ concurrently with AV-GBM-1, there was no difference in PFS curves by TMZ regimen administered concurrently (medians around 11 months), but OS was longer in patients who received concurrent TMZ alone (median 20.9 months) compared to TMZ plus bevacizumab (14.8 months) or TMZ + TTF (14.3 months) [35]. The inferior result for TMZ plus bevacizumab may be because these patients had cerebral edema and were felt to have PD or PsPD; so, bevacizumab was added to decrease the need for corticosteroids. The inferior result for TMZ + TTF may be because TTF potentially modified the cellular immune response in the GBM tumor bed, and more research on the effect of TTF on the tumor microenvironment is needed.

AV-GBM-1 has been approved for a double-blind, phase 3 trial with a 2:1 randomization of AV-GBM-1 to autologous monocytes with OS as the primary endpoint and subsequently modified into an adaptive phase 2/3 design (NCT05100641). Once again patients will be identified at the time of surgery for presumed GBM, then screened and randomized before concurrent RT/TMZ; treatment product will be manufactured during RT/TMZ, and patients will start study treatment after recovery from RT/TMZ with the first three injections given weekly, then concurrently every four weeks with a TMZ-based treatment of physicians’ choice, TMZ alone (preferred), TMZ plus bevacizumab (if needed to decrease use of corticosteroids), or TMZ plus TTF (discouraged based on the potential for TTF to affect an induced immune response). TTF was initially approved based on activity in patients with recurrent GBM [37], and investigators will be encouraged to reserve TTF for patients who have been taken off the study because of PD. Vaccine dosing will be at weeks 1, 2, 3, 8, 12, 16, 18, 24, 28, 36, 44, and 52 of year 1, and weeks 13, 26, 39 and 52 of years 2 and 3.
